# Scoping Review of EEG Studies in Construction Safety

**DOI:** 10.3390/ijerph16214146

**Published:** 2019-10-28

**Authors:** Yamei Zhang, Mingyi Zhang, Qun Fang

**Affiliations:** 1 School of Civil Engineering, Qingdao University of Technology, Qingdao 266033, China; zhangyamei@qut.edu.cn (Y.Z.);; 2 Department of Kinesiology, Mississippi State University, Mississippi State, MS 39762, USA

**Keywords:** EEG, mental status, safety management, construction industry, review

## Abstract

Construction safety is critical in the success of a project. A considerable amount of effort has been placed on research and practice in order to reduce the potential risks on the construction site. Recent application of electroencephalogram (EEG) to construction research enables researchers to gain insight into construction workers’ physical and mental status during construction tasks. By summarizing existing studies that involve EEG and construction safety, the literature review aims to provide practical suggestions for future research and on-site safety management. The literature search and inclusion process included eleven eligible studies. Comprehensive analysis was conducted based on primary and secondary measures. The primary measures considered the frequency bands of EEG and the channels for detecting electrical activity of the brain. The secondary measures that were involved with physical and mental status with respect to EEG signal variations as a result of task, working hour, and work conditions. Although the field of study that combines EEG measures with construction tasks is still emerging, it is worth continuous attention in the future, as relevant findings would be of great value to the safety management and risk control in the construction industry.

## 1. Safety Management in Construction Industry

Safety management and risk control have been major concerns in the construction industry. Construction workers are exposed to higher injury and death rates, two and three times respectively, than workers in the other industries [1]. A variety of models have been proposed to analyze potential reasons for on-site accidents. Sequential model attributes an accident to the cumulative consequence of a series of events and circumstances [2]. The model assumes that breaking the chain of accident evolution can prevent adverse outcomes from happening. Theoretically, with an increasing number of safety hazards being identified, risks that are exposed to the construction workers can be controlled at a minimal level. A recent study that was based on questionnaire surveys among 30 construction enterprises provided a systematic approach to reducing on-site risks. The research identified four critical aspects of construction safety management, including safety climate, safety culture, safety attitude, and safety behavior [3]. In addition, Choudhry and colleagues [4] proposed a similar but more detailed plan that consists of safety policy and standards, safety organization, safety training, hazardous conditions inspection, personal protection program, plant and equipment, safety promotion, and management behavior. Safety officers or project managers can make their construction sites safer by making endeavors to identify and exclude potential risks and hazardous conditions [4].

Behavioral models focus on human errors and dangerous behaviors that are considered the predominant sources of accidents, in contrast with the idea of making a thorough search for on-site risks and removing any hazardous factor from the sequence leading to an accident [2,5]. It has been found that reducing unsafe behaviors contributes to a significant improvement in workers’ safety performance [6,7]. In a previous study, behavior was defined as observable actions [6]. However, behavior is more than observable actions. It results from interactions among a combination of factors, such as physical workload, mental status, motivation, emotion, working environment, etc. Construction work is labor-intensive, which poses considerable challenges to workers’ physical and mental status [8]. Physical fatigue causes a decline in productivity and, notably, affects mental and psychological status, which increases workers’ vulnerability to hazards on the construction site [9]. Mental fatigue might result in depression, upset, boredom, frustration, and other negative emotions. Additionally, increased workload can cause inattentional blindness. When too much focus is placed on a complex task, people have less mental capacity to be aware of their surroundings [10]. Previous research summarized three major types of unsafe behaviors, including overlooking safety due to heavy workload, taking shortcuts to save effort and time, and inaccurate risk perception [11]. It is apparent that, as mental and physical exertion accrues during high-intensity physical efforts over long working hours, workers are more likely to conduct those unsafe behaviors [11].

The complexity of human behavior and the dynamic working environment have raised a series of challenges to current safety management. Traditional injury prevention strategies were effective between 1970s and the following 30 years [12]. However, safety performance in the construction industry failed to indicate significant improvement over the last decade, which suggests saturation in the effects of current injury prevention strategies and the need for innovations in safety management [13]. Automated technologies for real-time monitoring of workers’ behavior provide a promising approach to greatly improve the on-site safety management in the future [14,15]. Currently, the major form of on-site safety assessment is based on self-report questionnaire and safety checklist, which are considered to be cumbersome to implement on the construction site and prone to an individual’s subjective bias [16,17]. Therefore, a monitoring technique providing an objective assessment and real-time information with minimal intrusion to regular construction activities has great value in practice [14,17].

The value of real-time monitoring is to provide predictive information on workers’ behavior and potential risk on the construction site. Physiological parameters, such as hear rate, body temperature, oxygen consumption, and energy expenditure, are effective indicators of fatigue level, productivity, and physical workload in relation to an undertaking construction task [17,18,19,20]. In addition, position tracking systems, such as Ultra-Wide Band (UWB) and Global Positioning System (GPS), have been used to prevent workers from accessing danger areas [14,21]. As the risk level increases, a warning signal would be triggered to raise workers’ attention to potential risks in the surroundings [15]. Therefore, as compared with the traditional approach that relies on retrospective or lagging indicators, real-time monitoring shows a prominent advantage in on-site safety management [22].

Physiological measures are effective in reflecting workers’ physical exertion during construction activities, but a major challenge lies in monitoring workers’ mental and psychological state. EEG can provide direct and quantitative measures of the brain activity as a noninvasive neuroimaging technique [23]. The brain is involved with the initiation of voluntary movements. Therefore, brain activity detected by EEG is indicative of corresponding behaviors. Additionally, cognitive and mental status have significant influence on an individual’s behavior. Currently, EEG has been widely applied to examine cognitive performance, as fluctuations of EEG components can reflect the activation of cognitive systems [24]. The direct brain-behavior relationship implies that safety managers can identify workers that are exposed to increased risks by evaluating subtle changes in their EEG-based behavioral and cognitive status during construction works. With the assistance of EEG technique, necessary procedures can be undertaken in advance to prevent unsafe behaviors and make on-site risks under control. EEG has been considered to be the best neuroimaging technique for construction implementation because of its advantages in mobility, portability, and tolerance to movement [8,10,25,26]. Researchers even expect a revolutionary change in construction safety by means of the EEG-based monitoring technique [10]. Given the promising effect of EEG in safety enhancement, researchers and practitioners have shown increased interest in applying EEG to construction sites. The current literature review summarized existing studies concerned with EEG application to the construction context. We aim to provide suggestions for future research as well as for safety management practices in the construction industry by searching and reviewing relevant studies in a systematic approach.

## 2. Literature Search

### 2.1. Search Strategy

The literature search was conducted through PubMed, IEEE, EMBASE, EBSCO, and Web of Science. Databases in the field of neuroscience and construction engineering are selected because the current review involves research applying EEG to construction tasks. PubMed and EMBASE are important sources for health, neuroscience, and biomedical topics, while IEEE is selected for literature on construction and engineering. In addition to the databases for specific academic fields, EBSCO and Web of Science provide access to a general search. By using multiple databases, the authors aim to conduct a thorough search for relevant studies. The range of year for literature search is between 2000 and 2019. Given that the focus of the current review is placed on EEG and its application to safety management in the construction-related tasks, the search scope considers four main aspects: Construction workers, safety, physical or mental performance, and EEG. Accordingly, the following combinations of key terms were used for literature search: “construction workers” and “safety management OR risk control” and “physical exertion OR fatigue OR mental workload OR attention OR emotion” and “electroencephalogram OR EEG.”

To determine the eligibility of each identified study for inclusion, several criteria were pre-determined before (1) peer-reviewed articles published in English; (2) construction workers were subjects of a study; (3) an experiment must involve on-site work conditions or construction tasks; and, (4) real-time EEG data should be recorded as subjects were performing construction activities. The current review only considers experimental studies published in peer-reviewed journals to provide empirical evidence and suggestions for future research as well as practical safety management. Thus, the review paper, book chapter, and conference abstract will be excluded.

### 2.2. Study Selection

A two-phase screening process was performed to select studies for inclusion. The initial phase removed duplicates and irrelevant articles based on title and abstract examination. In the second phase of screening, a full-text evaluation was conducted to assess the eligibility of the remaining articles. In addition to the database search, a snowball search was conducted in the second phase. During the full-text evaluation, a few references that were cited by the articles in the second phase appear to fit into the interest of the current review. Full-text evaluation on the eligibility of the references was then performed along with the other studies in the second phase of the screening process. Two researchers (YZ and QF) worked independently to identify relevant papers. Any disagreement on the eligibility of an article was resolved by having a discussion with another author (MZ) during consensus meetings.

**Figure 1:**
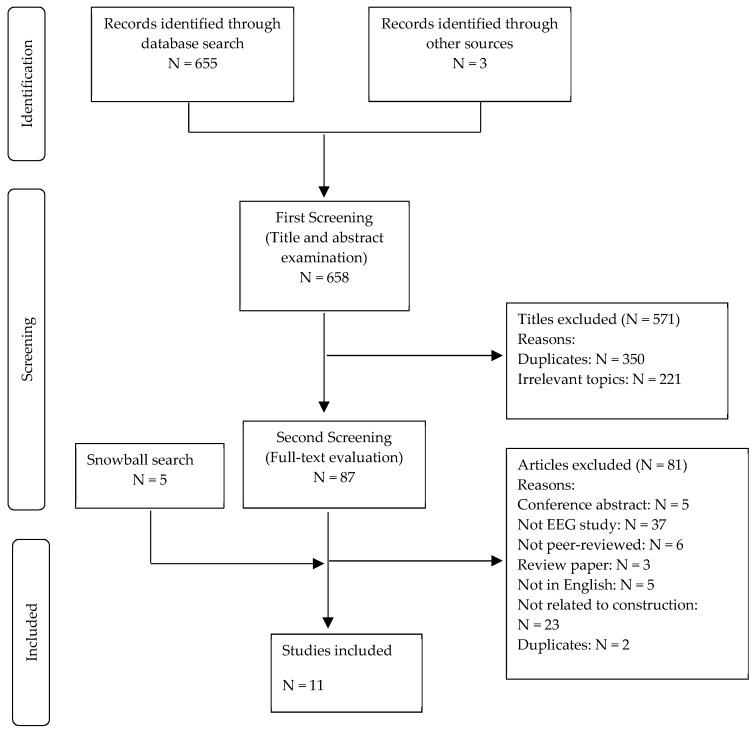
Flowchart of the inclusion process.

An initial search across the five databases resulted in a total of 655 articles. Another three articles were added to the initial search from other sources (e.g., expert’s advice). Of the 658 articles that were identified in the initial search, 571 articles were excluded in the first phase of screening because of duplicates (N = 350) and irrelevant topics (N = 221). There were 92 articles that were considered for full-text analysis, with five references identified from the snowball search. The second screening phase removed 81 articles for the following reasons: conference abstract (N = 6), review paper (N = 3), not using EEG (N = 37), not peer-reviewed article (N = 6), duplicate articles (N = 2), not related to construction task (N = 23), and not written in English (N = 5). The screening process finally retained 11 articles for quantitative review. Figure 1 shows each step in the selection process.

### 2.3. Data Extraction

Study characteristics, such as publication year, sample size, and apparatus used in the studies, will be summarized to provide basic information of the included articles. Construction work impacts an individual’s emotion, cognitive function, and mental state. Therefore, one major issue that is addressed in the current review is to find out how mental and physical state can be quantified by EEG components. Information regarding EEG channels (e.g., AF3, F3, etc.), frequency bands (e.g., theta, alpha, beta, etc.), and power spectral density will be collected from the included studies, which may offer valuable information for the subsequent research. Another important concern of the current review is about the changes of EEG signals in relation to construction tasks, work conditions, and working hours. A summary of findings that are related to the second issue would be helpful in interpreting EEG signals, thus providing great value for safety management practice.

## 3. EEG Measures in Relation to Construction Activity

### 3.1. Study Characteristics

According to the year of publication, all of the included studies were published in the last three years (2016–2019), with one publication in 2016 [10], five publications in 2017 [16,17,25,27,28], three publications in 2018 [8,29,30], and two publications in 2019 [31,32]. The information suggests a trend that EEG-based research on safety and risk management is an emerging topic that is growing fast and drawing increasing attentions from researchers. Sample size of the included studies ranged between five and 30, with a median of 10 subjects in the studies. There are two EEG models applied to the included studies. One commonly used model is EPOC+, with 14 channels and two reference electrodes–P3 and P4 locations according to the 10–20 system [8,16,25,28,29,30,31,32]. Three of the included studies employed the NeuroSky EEG headset for neural signal collection [10,17,27]. The included studies investigated frequency band variations in relation to mental and physical functions and mainly focused on two main aspects: (1) the frequency band applied to measure brain activities associated with the above-mentioned functions; and, (2) the electrodes that were used to detect EEG signals with respect to each function. Further details regarding the characteristics of the included studies can be found in Table 1.

The prominent advantage of EEG is a direct measurement of neural activities that reflect cognitive dynamics over time [10,24]. The primary measures take into account various frequency bands, including delta (<4 Hz), theta (4–7 Hz), alpha (8–13 Hz), beta (14–30 Hz), and gamma waves (>30 Hz) [33]. The delta wave is associated with a state of unconsciousness, such as deep sleep. The theta wave is related to drowsiness, inattention, and meditation. The alpha wave reflects physical and mental relaxation with awareness of one’s surroundings. The beta frequency band is predominant in the states of alertness, concentration, anxious thinking, tension, and fear. The gamma waves can be seen in mentally demanding activities and information processing, such as learning and problem solving [34].

### 3.2. EEG Measures of Risk Perception

Based on the features of each type of brainwave, the mental status of construction workers was assessed by the ratios and indices of EEG components. Engagement index Equation (1) is a measure of mental workload, which is computed by the power spectral density (PSD) of theta, alpha, and beta waves [10]. The PSD represents the power distribution of EEG components in the frequency domain [16]. The unit of PSD is usually denoted as microvolts-squared per Hz (mV^2^/Hz). In the research that was conducted by Chen and colleagues, EEG data were collected from four sites, including two prefrontal areas (FP1 and FP2) and two reference points after ears (TP 9 and TP 10). While FP1 reveals logical attention, such as decision making and working memory, FP2 provides information on emotional attention, such as stress, impulse, and judgment [10,27]. The engagement index indicates a positive relationship to the level of mental workload. Beta (14–30 Hz) frequency becomes dominant when a person indicates a high level of alertness. Therefore, an increase in the index is a sign of more attentions paid to an ongoing task, which implies the reduced capacity of detecting potential risks on the construction site.

(1) $$\mathrm{Engagement}\;\mathrm{index}=\frac{\beta }{\theta +\alpha }$$

Vigilance reflects an individual’s attention level when performing a task. Construction workers with high vigilance are able to stay alert during the task and make quick responses to an approaching hazard. However, when mental workload increases along with performing a complex task, vigilance level with respect to surrounding risks decreases. As a result, construction workers are more likely to underestimate the surrounding risks and adopt unsafe behaviors [10]. In an experiment consisting of six construction tasks, researchers assessed subjects’ vigilance stage when performing different tasks [16]. The study identified that the gamma wave (30–40 Hz) and the left frontal channel clusters (AF3, F7, and F3) were the suitable frequency band and brain regions to provide valid measures of vigilance. However, more comprehensive ratio indices comprising various frequency bands have been proposed because a single frequency band is difficult to directly measure changes of vigilance [35]. A following research investigated validity of 30 indices assessing vigilance level of the construction workers, with three indices indicating the feasibility of being applied to in vigilance detection [32]. The theta/beta ratio Equation (2) results from dividing the short-wave power density (theta wave) by fast-wave power density (beta wave) [36]. A relative predominance of the beta wave lowers the ratio, which suggests the better functioning in orienting network—directing attention to a target stimulus [37].

(2) $$\mathrm{Vigilance}=\frac{\theta }{\beta }$$

In addition, two author-designed indices were found to be strongly correlated to vigilance Equations (3) and (4). The designed indices comprise high frequency band, such as gamma wave (30–50 Hz). The high correlation coefficients validate the two quantitative vigilance indicators. However, as the researchers discussed in the article, more experiments are needed to explain the high correlation among the three indicators [30].

(3) $$\mathrm{Vigilance}=\frac{\theta +\beta }{\alpha +\gamma }$$

(4) $$\mathrm{Vigilance}=\frac{\alpha }{\beta +\gamma }$$

### 3.3. EEG Measures of Emotional Status

Emotional status involves two dimensions, including valence, varying from negative to positive, and arousal, varying from low to high [38]. While an individual’s behavior is indicative of his or her emotional states, emotional states are also predictive of one’s behavioral outcomes [39]. Real-time monitoring of emotional status is important in preventing construction workers from unsafe behaviors due to the strong relationship between emotion and behavior. The commonly used measure of valence is based on the approach/withdraw model of frontal EEG asymmetry, which states that the relatively greater left frontal activity corresponds to affectively positive stimuli, whereas increased right frontal activity or reduced left frontal activity corresponds with affectively negative stimuli [40,41]. The included studies of the current review adopted alpha and beta rhythms in the bilateral frontal lobe (AF3, F3, AF4, and F4) to quantify the variations of emotional status and arousal level during construction activities [8,28]. However, the two measures are analyzed in different ways. Where valence is calculated by asymmetric activities between left and right hemisphere Equation (5), arousal is the ratio of alpha to beta rhythm in the frontal regions of both hemispheres Equation (6). A positive valence is indicative of greater activity in the left hemisphere, which suggests more pleasant emotions [8,26].

(5) $$\mathrm{Valence}=\frac{\alpha \left(F4\right)}{\beta \left(F4\right)}-\frac{\alpha \left(F3\right)}{\beta \left(F3\right)}$$

(6) $$\mathrm{Arousal}=\frac{\alpha \left(AF3+AF4+F3+F4\right)}{\beta \left(AF3+AF4+F3+F4\right)}$$

In the included studies, valence and arousal are both calculated based on the power spectral density (mV^2^/Hz) of alpha and beta bands in the frontal areas. According to the approach/withdraw model, greater activity in the left hemisphere is associated with an approach-related effect to positive emotions [42]. Given the feature of alpha and beta frequency that is characterized by relaxation and active state, respectively, increased activation in the left hemisphere is represented by relatively predominant power density of beta frequency in AF3 and F3 [8]. Therefore, a greater valence implies positive emotions, such as joy and happiness. Additionally, the ratio of the mean power spectral density of alpha to that of beta is a measure of arousal state [8,28]. A greater value of the alpha/beta ratio in the frontal areas indicates more aroused emotional state, such as alertness and excitation [8].

### 3.4. EEG Measures of Physical and Mental Fatigue

Beta rhythm (12–30 Hz) has been found to be a suitable indicator of physical exertion [25,27]. Chen et al. mentioned that the beta band is useful in identifying less mentally demanding, but more physically demanding tasks [27]. Body movement is associated with motor cortex activation, which can be detected by electrodes of FC5 and FC6 [25]. The power spectral density of beta frequency becomes predominant as physical exertion increases. Therefore, the EEG signals differentiate physically active conditions from inactive conditions [27]. Researchers attempt to investigate whether physical fatigue as a result of prolonged physical activity is associated with a decline in the power density of beta frequency based on the findings of the positive relationship between physical exertion and the power of beta rhythm. However, the only included study that considered this issue did not find adequate evidence [17]. The study measured physical fatigue by means of both EEG ratio and Borg’s Rating of Perceived Exertion (RPE), which reflected participants’ perceived fatigue level from 1 (low fatigue level) to 4 (very high level) [43]. The EEG measure of fatigue is based on the ratio represented by Equation (7), which suggests that a greater ratio is a sign of increased fatigue level. A positive relationship is assumed between the EEG ratio and the RPE score. However, as the EEG ratio showed inconsistent changes among the participants, no conclusive evidence was found to substantiate the assumption [17].

(7) $$\mathrm{Fatigue}\;\mathrm{level}=\frac{\alpha +\theta }{\beta }$$

Mental fatigue is another prominent risk factor in the construction site. Li and colleagues examined the effects of four EEG ratios on reflecting individual’s mental fatigue [31]. The indicators comprise power spectral density of theta, alpha, and beta frequencies. By considering the four indicators as well as other influential factors on mental state, including self-reported fatigue level, sleep hours, and performance in the Stroop test, researchers found two indicators, Equations (7) and (8) to be effective in identifying workers with high mental fatigue levels.

(8) $$\mathrm{Fatigue}\;\mathrm{level}=\frac{\theta }{\beta }$$

Theta wave is indicative of inattention, distractibility, and depression [27]. The power spectral density of theta frequency tends to become predominant due to accumulated fatigue. In addition, as an indicator of mentally and physically active status, beta frequency can be depressed due to the increased fatigue. Therefore, an increased value of the ratios implies greater risks associated with fatigue. Based on EEG spectral analysis, the researchers developed an algorithm to screen workers with a high fatigue level [31]. Project managers may use such a quantitative assessment instrument to identify individuals who may not be qualified to perform the construction tasks demanding high mental and physical workload.

### 3.5. EEG Measures in Relation to Construction Activities

Task allocation influences construction workers’ mental status, such as arousal, attention, and motivation, which are critical factors in safety management [27]. Managers should consider impacts of construction tasks on workers’ mental status to make appropriate decisions on task allocation. After reviewing the measures of mental, emotional, and physical states employed in the included studies, we pay attention to the impacts of working conditions and construction activities on EEG signals. Working conditions involved working at ground level, on top of a ladder, and in a confined space [8,28]. Construction activities include ladder climbing, nuts selection, bolts fastening, and other normal work on the construction sites [10,25,27]. The included studies identified altered EEG patterns as a function of work conditions and tasks. Analysis in the following sections focused on the impacts of work conditions, tasks, and working hours on mental status, as reflected by variations in EEG signal.

#### 3.5.1. Work Conditions

Four of the included studies investigated workers’ emotional status in different working conditions [8,28,29,30]. The subjects in the two studies were asked to perform construction tasks at the ground level, on top of a ladder, and in a confined space. The results indicated that working in a confined space and on a top of a ladder led to undesirable emotions that were observed as an under-activation in the beta frequency band of the left frontal lobe. In contrast, working at the ground level suggested a neutral to positive influence on workers’ mental status. The researchers also assessed the effects of chatting and short rest during the construction tasks. Chatting with coworkers reduced negative emotions, as evidenced by an increased valence value from −1.39 to −0.11 [28]. In addition, taking a short break during work indicated a positive effect on workers’ emotions [8,28].

#### 3.5.2. Tasks

Chen and colleagues examined mental workload in relation to different tasks [10,27]. Both studies examined engagement index Equation (1) and the power of gamma rhythm when subjects were performing construction related tasks, including ladder climbing, nut selection, and bolt fastening. Signal spikes in the engagement index and the gamma band were observed in ladder climbing and bolt fastening, which suggested a higher level of mental demanding than nut selection. In another study regarding vigilance and attention level, subjects needed to avoid obstacles while carrying heavy objects to an assigned place [16]. A strengthened power of gamma rhythm was observed when construction workers were facing obstacles on the way. The EEG signals suggest increased attention level in response to the potential hazards.

#### 3.5.3. Working Hours

The emotional status of construction workers varies as a function of working hours. Hwang et al. conducted an experiment in which EEG signals recorded mental states at three time points: after rest, 1 h after rest, and 2 h after rest [8]. A short break resulted in reduced arousal and a positive valence, which suggested a state of contentment and relaxation. Working 1 h after rest appears to generate the most desirable emotional status, with happiness and joy being reflected in the EEG signals. Negative emotions can be identified in the situation of working 2 h without rest. Construction workers indicated a combination of negative valence, but positive arousal, which was associated with frustration and anger [8].

## 4. Discussion

Safety management and risk control has been an important issue in the construction industry [16]. Construction workers are exposed to potential risks and unexpected hazards during their ongoing tasks, especially when they are performing a task of considerable mental and physical demand. Therefore, monitoring construction workers’ physical and mental status would be of great value in keeping on-site risks under control. Instruments that are based on self-report survey and biofeedback (i.e., skin temperature and heart rate) have been widely applied to construction sites. However, it has been a challenge to reflect an individual’s mental and psychological condition through objective and reliable means [10,16]. Recently, a wearable EEG device has largely overcome technical limitations and been applied to relevant research. In the included studies, EEG-based algorithms (i.e., online multi-task learning algorithms) have shown reasonable accuracy in identifying individuals at high risks due to increased stress and fatigue level during construction tasks [29,30]. The current literature review included studies that applied EEG to monitor construction workers’ physical and mental status during construction tasks. All of the studies confirmed the validity of using EEG to assess the physical and mental status of construction workers. The groundwork has shown a promising direction of integrating neuroscience into the study of safety management in the construction industry.

EEG analysis involves the computing power of frequency bands, including delta, theta, alpha, beta, and gamma, or ratios between these frequency bands [44]. Different frequency bands of EEG signals can reflect the mental and psychological status, including mental workload, valence, arousal, and vigilance. Gamma band is mainly associated with perceptual processes [45]. Cognitive functions, such as attention [46,47], arousal [48], object recognition [49,50], and language perception [51], can be identified within the gamma frequency range. Previous research on industrial and occupational fatigue mostly used low frequency bands, such as theta and alpha waves [52,53,54]. However, the included studies of the current review recognized gamma activity during construction work, which suggested that construction tasks are more than labor-intensive and manual in nature [10,16,27]. Beta band is predominant in an alert and active state [34,55]. An increase in the beta band is often associated with a physically active state as well as a high level of alertness, motivation, emotion, and mental activity [54]. Therefore, an increase in the engagement index can be observed with increased mental workload as well as the attention level during construction tasks [10,16,27]. On the other hand, reduced beta power density could be a sign of less active status related to fatigue. Evidence can be found in the fatigue indices, in which the beta wave is the denominator Equations (7) and (8). Reduced beta power density inflates the indices, which suggests increased fatigue level [31]. Alpha and theta are at relatively low frequency ranges that reflect an inactive and relaxed state. Alpha and theta activity tend to be suppressed with higher level of concentration, whereas increase as a sign of fatigue or reduced alertness, such as drowsiness, boredom, and exhaustion [31,56]. Alpha and theta frequency bands are sensitive measures of fatigue [57]. The included studies identified the tendency of the declined engagement index as a result of accumulated mental fatigue during prolonged construction work [10,27].

Brain region is another main consideration in EEG data analysis. Frontal regions (FP1, FP2, AF3, F3, AF4, and F4) are the primary areas to detect mental status, because the frontal and prefrontal cortex are responsible for important cognitive functions, such as emotional expression, problem solving, reasoning, decision-making, and executive functions [58]. Emotional state is often measured by cortical activities between the left and right frontal lobe [8,28]. It is worth noting that the activation of the left frontal areas or deactivation of the right frontal areas is associated with positive emotions, such as happiness, joy, and contentment [8]. Brain activities that are related to physical exertion can be detected based on the EEG signals in the motor cortex (FC5 and FC6). Electrodes placed in the area of FC5 and FC6 can indicate the difference between the active and inactive conditions [25].

Practical advice can be developed from the findings of the included studies. Construction workers may need to avoid working in an uncomfortable or dangerous site (i.e., in confined space or at height) over 2 h due to the impacts of working hours and conditions on valence, arousal, attention, and vigilance [8,28,29,30]. It would be necessary to divide a complex and time-consuming project into multiple intervals to provide workers with adequate rests. Therefore, diverse working hours and working conditions could help construction workers to keep a positive emotional status and high arousal level [8]. Current studies found that mentally demanding tasks reduce perception capacity, which makes the construction workers vulnerable to unexpected hazards [10]. In accordance with the finding, necessary precautions, such as trip and fall protection, electric shock prevention, and falling objects protection, must be implemented in the areas where complex tasks are performed [59]. The abovementioned suggestions for on-site safety management have already been known to the construction industry, but it is the first time that the safety procedures are substantiated by neuroscience evidence.

## 5. Limitations and Future Direction

A major limitation of the current review is the small number of studies, given that only eleven articles met the eligible criteria. In addition, the sample size is also small, which can be largely attributed to the fact that primary purpose of the preliminary studies is to test the validity and reliability of using EEG to monitor construction workers’ physical and mental status during work. Another limitation is related to the heterogeneity across the included studies, with three studies focusing on the fatigue level of construction workers [17,25,31], four studies examining emotion and motivation [8,28,29,30], and four studies investigating mental workload [10,16,27,32]. Diverse focuses of these studies provide a broad view on EEG patterns and construction activities, but further investigations are needed in the future research. Researchers and practitioners should interpret results of the review with caution because of the limitations in the current review.

Despite the limitations at this moment, preliminary studies have shown a promising direction for construction safety research in the future. The application of EEG provides an additional dimension of safety control in the construction industry [10]. Brain activities in response to different tasks and work conditions largely remain unknown because relevant research in this field is still at an initial phase. For example, the tasks that are involved in the reviewed studies (i.e., bolts fastening and ladder climbing) only account for a limited proportion of construction workers’ daily routines. It would be valuable to investigate physical and mental status under various situations. In addition to safety management, the productivity of construction workers could be another interest of EEG study. Researchers have applied EEG to monitor workers’ attention level, emotion, and motivation, which are essential factors with respect to productivity in the workplace. With the knowledge of desirable emotional and mental status, appropriate task allocation can be assigned to optimize the performance and productivity of construction workers [27].

The application of neuroscience to construction engineering will not only improve safety management practice, providing refined techniques in automated assessment and prediction of construction workers’ behaviors, but also benefit neuroscience in return. It is interesting to see such a fast development in the past few years, during which researchers attempted to validate the method of using wearable EEG to reflect construction workers’ physical and mental changes in both lab and on-site environment in the first two years between 2016 and 2017 [10,16,27,28]. However, recent publications in the last two years indicate a trend toward acquiring high quality of EEG signals and improving data processing techniques by means of algorithms [29,30,31,32]. Neuroscience enables scientists in the civil engineering to understand an individual’s behavioral and psychological responses from the neural level. In the meantime, the algorithms that were developed from engineering will provide neuroscientists with powerful research instruments to filter artifacts due to movement and explore brain activities in dynamic, movement-related conditions that were difficult to study without effective and robust information processing techniques. The interdisciplinary benefits between neuroscience and civil engineering will contribute to continuous advancement in both fields.

## 6. Conclusions

EEG is a promising approach for improving the effectiveness of safety management on the construction site. The included studies substantiate the feasibility of using EEG technique as a measure of construction workers’ physical exertion, mental workload, and emotional status. In addition, EEG signal variations in relation to different construction tasks, working hours, and work conditions provide predictive information on construction workers’ behaviors. The current review summarizes recent findings based on combined research work between civil engineering and neuroscience. Researchers in the civil engineering may use the current review to develop and refine EEG-based experiments, and thus contribute to enhancing on-site safety management in the construction industry. On the other hand, neuroscientists are able to acquire high-quality EEG data from the experiments requiring body movement by taking advantage of the artifact removal techniques. Future research is needed to expand the current knowledge base on the relationship between EEG patterns and corresponding construction activities, which enables practitioners to optimize the performance of construction workers while maintaining on-site risks under control.

## Figures and Tables

**Table 1:** Summary of study characteristics.

Study	Sample Size	Apparatus	Primary Measures	Secondary Measures	Main Findings
Chen et al., 2016 [10]	N = 5	NeuroSky Think Gear	Frequency bands: alpha, beta, and theta; EEG channels: FP1 and TP10. Engagement index = $\frac{\beta }{\alpha +\theta }$	Mental workload in various construction tasks (ladder climbing, nuts selection, and bolts fastening)	Power spikes of engagement index can be seen in the process of ladder climbing and bolt fastening, which suggest lower risk perception ability and higher risk for accidents during the tasks.
Wang et al., 2017 [16]	N = 10	EPOC+ 14 Channels	Frequency bands: alpha, beta, and gamma waves; EEG channels: Left cluster (AF3, F7, and F3).	Vigilance NASA-TLX scores	Vigilance of construction workers is related to different tasks, which can be measured by EEG frequency bands and channels. The gamma frequency bands and left frontal channel clusters (AF3, F7, and F3) can reflect vigilance variations in EEG signals.
Aryal et al., 2017 [17]	N = 12	NeuroSky MindWave 2	Frequency bands: alpha, beta, and theta; Mental fatigue = $\frac{\alpha +\theta }{\beta }$	Physical fatigue monitored by skin temperature and heart rate	The ratio showed some increase along with the development of physical fatigue. However, no consistent changes were observed in the EEG signal among the participants.
Jebelli et al., 2017 [25]	N = 8	EPOC+ 14 Channels	Frequency bands: beta; EEG channels: Motor cortex area (FC5 and FC6).	Physical exertion—Use EEG to differentiate physically active state from inactive state	Higher spectral power of the beta frequency band is associated with physical activities in construction tasks compared with inactive condition.
Chen et al., 2017 [27]	N = 30	NeuroSky Think Gear	Frequency bands: alpha, beta, and gamma; EEG channels: FP1, FP2, TP9, and TP10.	Mental workload in various construction tasks (ladder climbing, nuts selection, and bolts fastening)	Mental workload can be reflected in EEG signals. In comparison with the alpha and beta bands, high-frequency gamma band is more suitable for task differentiation and is positively related to the mental demand.
Jebelli et al., 2017 [28]	N = 8	EPOC+ 14 Channels	Frequency bands: alpha and beta; EEG channels: Frontal clusters (AF3, F3, AF4, and F4). Valence = $\frac{\alpha \left(F4\right)}{\beta \left(F4\right)}-\frac{\alpha \left(F3\right)}{\beta \left(F3\right)}$	Emotions in relation to various real work conditions (working at ground level, top of the ladder, and in confined space)	The valence index is negative with respect to working on top of the ladder and in a confined space, which suggests negative emotional states under the two work conditions.
Hwang et al., 2018 [8]	N = 10	EPOC+ 14 Channels	Frequency bands: alpha and beta; EEG channels: Frontal clusters (AF3, F3, AF4, and F4). Valence = $\frac{\alpha \left(F4\right)}{\beta \left(F4\right)}-\frac{\alpha \left(F3\right)}{\beta \left(F3\right)}$ Arousal = $\frac{\alpha \left(AF3+AF4+F3+F4\right)}{\beta \left(AF3+AF4+F3+F4\right)}$	Emotional state—valence and arousal—in relation to working conditions (working at ground level, on top of a ladder, and in a confined space) and hours (working after rest, 1 h, and 2 h)	Workers working at ground level for 1 h after rest display positive valence and arousal which imply positive emotions such as happiness and joy. Working in a confined space or at height for 2 h results in frustration and reduced alertness.
Jebelli et al., 2018 [29]	N = 7	EPOC+ 14 Channels	Frequency bands: alpha and beta; EEG channels: Frontal clusters (AF3, F3, AF4, and F4); Stress level based on EEG signal.	Cortisol level (a measure of stress) in relation to various real work conditions	EEG-based stress recognition, online multi-task learning algorithms (OMTL), indicated high accuracy of predicting new stressful situations in both lab environment and real construction sites.
Jebelli et al., 2018 [30]	N = 7	EPOC+ 14 Channels	Frequency bands: alpha and beta; EEG channels: Frontal clusters (AF3, F3, AF4, and F4); Stress level based on EEG signal.	Cortisol level (a measure of stress) in relation to various real work conditions (working at ground level, top of the ladder, and in confined space)	EEG signals based on the fixed windowing approach and the Gaussian Support Vector Machine indicated the highest classification accuracy (80.32%) of stress identification.
Li et al., 2019 [31]	N = 15	EPOC+ 14 Channels	Frequency bands: theta, alpha, and beta; Mental fatigue level is calculated by four EEG-based indicators.	Mental fatigue level EEG indicators Self-reported fatigue Stroop test	EEG indicators are effective in assessing mental fatigue level and filtering construction workers who are not qualified for the on-site work due to mental fatigue.
Wang et al., 2019 [32]	N = 10	EPOC+ 14 Channels	Frequency bands: alpha, beta, and gamma waves; EEG channels: All 14 channels of the device. Vigilance was measured by candidate indices.	Vigilance NASA-TLX scores	Among 30 candidate indices of vigilance, three indices showed highest correlation to construction workers’ vigilance.
